# Cullin4 E3 Ubiquitin Ligases Regulate Male Gonocyte Migration, Proliferation and Blood-Testis Barrier Homeostasis

**DOI:** 10.3390/cells10102732

**Published:** 2021-10-13

**Authors:** Yan Yin, Liming Zhu, Qiufang Li, Pengbo Zhou, Liang Ma

**Affiliations:** 1 Department of Medicine, Division of Dermatology, Washington University School of Medicine, 660 S. Euclid Avenue, St. Louis, MO 63110, USA; yanyin@wustl.edu (Y.Y.); zhuliming1971@outlook.com (L.Z.); liqiufang0811@126.com (Q.L.); 2 Department of Pathology and Laboratory Medicine, The Joan and Stanford I. Weill Medical College of Cornell University, New York, NY 10021, USA; pez2001@med.cornell.edu

**Keywords:** ubiquitination, Cullin4, spermatogenesis, blood-testis barrier

## Abstract

Ubiquitination, an essential posttranslational modification, plays fundamental roles during mammalian spermatogenesis. We previously reported the requirement of two Cullin 4 ubiquitin ligase family genes, Cullin 4a (*Cul4a*) and Cullin 4b (*Cul4b*), in murine spermatogenesis. Both genes are required for male fertility despite their distinct functions in different cell populations. *Cul4a* is required in primary spermatocytes to promote meiosis while *Cul4b* is required in secondary spermatocytes for spermiogenesis. As the two genes encode proteins that are highly homologous and have overlapping expression in embryonic germ cells, they may compensate for each other during germ cell development. In the present study, we directly address the potential functional redundancy of these two proteins by deleting both Cul4 genes, specifically, in the germ cell lineage during embryonic development, using the germ-cell specific *Vasa*-Cre line. Conditional double-knockout (dKO) males showed delayed homing and impaired proliferation of gonocytes, and a complete loss of germ cells before the end of the first wave of spermatogenesis. The dKO male germ cell phenotype is much more severe than those observed in either single KO mutant, demonstrating the functional redundancy between the two CUL4 proteins. The dKO mutant also exhibited atypical tight junction structures, suggesting the potential involvement of CUL4 proteins in spermatogonial stem cell (SSC) niche formation and blood–testis-barrier (BTB) maintenance. We also show that deleting *Cul4b* in both germ and Sertoli cells is sufficient to recapitulate part of this phenotype, causing spermatogenesis defects and drastically reduced number of mature sperms, accompanied by defective tight junctions in the mutant testes. These results indicate the involvement of CUL4B in maintaining BTB integrity.

## 1. Introduction

Male infertility, a major issue in reproduction, affects approximately one in 25 men in the US [1]. Although its etiology is heterogeneous, it is usually associated with oligozoospermia (low sperm count), asthenozoospermia (poor sperm motility), and teratozoospermia (abnormal sperm morphology). Mammalian spermatogenesis is a tightly orchestrated and dynamic process that transforms pluripotent spermatogonia into mature gametes or spermatozoa in three distinct phases: mitosis, meiosis, and spermiogenesis. During mouse embryonic development, sexually undifferentiated primordial germ cells (PGCs) formed in the proximal epiblast migrate toward the genital ridges at E7.5, where they become enclosed by the somatic Sertoli cells to form seminiferous cords at E12.5–E13.5 [2]. While migrating, PGCs undergo rapid proliferation, and those arriving late or migrating to the wrong place are eliminated by apoptosis [3]. At this stage, PGCs are called gonocytes and they continue to proliferate until they enter quiescence, at around E16.5 [4]. Around birth, gonocytes start to migrate from the lumen of the seminiferous tubules towards the basement membrane, and resume proliferation to give rise to spermatogonial stem cells (SSCs) [5]. Sertoli cells provide essential extrinsic factors including binding and transport proteins, protease and protease inhibitors, hormones, and growth factors (for review see [6]), and establish a permissive niche to direct gonocyte migration and facilitate their differentiation into SSCs [7]. The SSCs have the capacity to self-renew as well as to become proliferative progenitor spermatogonia that eventually undergo spermatogenesis.

Maintenance of the SSC niche and successful spermatogenesis rely not only on germ cells themselves, but also architectural support and regulatory factors provided by the somatic cells, as well as reciprocal germ-somatic cell interactions. Inside the testis, tight junctions (TJs) and other junction structures located on adjacent somatic Sertoli cells, commonly known as the blood–testis barrier (BTB), physically divide the seminiferous epithelium into basal and adluminal compartments [8]. SSCs and differentiated spermatogonia reside inside the basement compartment. Once preleptotene spermatocytes emerge after a series of mitotic divisions, they are ready to traverse the BTB for entry into the adluminal compartment to complete two consecutive rounds of meiosis and subsequent spermiogenesis. Defects that occur in either somatic or germ cells during this highly complicated process can lead to compromised male fertility.

The rapid and tightly regulated progression of spermatogenesis depends on both the proteolytic as well as the non-proteolytic actions of protein ubiquitination [9,10]. The ubiquitination of proteins occurs through the hierarchal actions of ubiquitin-activating, -conjugating, and -ligating enzymes, and substrate specificity is determined by the E3 ubiquitin ligases. We have previously studied the functions of the two members of the vertebrate Cullin–RING finger ligase 4 (CRL4) family, Cullin4a (CUL4A) and Cullin4b (CUL4B), in mammalian spermatogenesis. Null mutation of *Cul4a* leads to male infertility characterized by oligozoospermia, asthenozoospermia, and teratozoospermia, due to meiotic progression defects at the pachytene stage and subsequent germ cell death [11]. The conditional removal of *Cul4b* in germ cells results in male infertility associated with asthenozoospermia and teratozoospermia due to impaired spermiogenesis. In addition, global deletion of *Cul4b* leads to an age-dependent germ cell depletion phenotype, implicating its role in maintaining the SSC niche [12]. In the current study, we continue to explore the roles of CUL4 E3 ubiquitin ligases in mammalian spermatogenesis by demonstrating their essential functions in germ cell migration, survival as well as in maintaining BTB integrity.

## 2. Materials and Methods

### 2.1. Mice

The generation of *Cul4a* and *Cul4b* floxed allele mice was described previously [12,13]. *Vasa*(*Ddx4*)-Cre (stock# 006954) and *Amh*-Cre (stock#007915) transgenic mice were purchased from the Jackson Laboratory (Bar Harbor, ME, USA). The morning upon which a vaginal plug was detected was considered embryonic day 0.5 (E0.5) of pregnancy, and embryos of desired embryonic age were harvested accordingly. All mice were maintained in a barrier facility at Washington University School of Medicine, and all animal experiments were performed in accordance to the institution’s regulations with an approved protocol (#20190040).

### 2.2. Histology, Immunofluorescence (IF) and TUNEL Assays

Freshly dissected testicular/epididymal tissues were fixed in Bouin’s fixative (H&E, IF) or 4% paraformaldehyde (TUNEL) overnight at 4 °C, and processed for paraffin embedding following routine procedures. IF and TUNEL assays were performed as described previously [11]. The antibodies and dilutions used were: 1:100 for CUL4B (#20882-1-AP, Proteintech Group, Chicago, IL, USA) and CUL4A (#IHC-00145, Bethyl Laboratories, Montgomery, TX, USA); 1:200 for AR (#sc-816, Santa Cruz Biotechnology, SCBT, Dallas, TX, USA) and SCP3 (#sc-74569, SCBT); 1:500 for VASA (#8761, Cell Signaling Technology, CST, Danvers, MA, USA), CTNNB1 (#610154, BD Biosciences), pS6S235/236 (#2211, CST), pS6S240/244 (#2215, CST), and CLDN11 (#36–4500, ThermoFisher, Waltham, MA, USA); 1:1000 for Alexa 594 goat anti-rabbit, Alexa 488 goat anti-mouse and Alexa 488-conjugated lectin PNA (ThermoFisher). IF images were captured under a Zeiss AxioSkop 2 fluorescent microscope. Confocal microscopy was imaged on Zeiss LSM 880 Airyscan at the Washington University Center for Cellular Imaging.

### 2.3. Western Blotting

Freshly dissected testes were decapsulated and homogenized in 1× RIPA buffer containing protease inhibitor cocktail, sonicated, and supernatant collected. Protein concentrations were determined by Bradford assay [14]. Laemelli buffer was added to tissue lysates and boiled for 5 min to denature proteins. A volume of 30 µg of total proteins per sample were separated on 4–15% Bis-Tris gels and electrophoretically transferred to PVDF membranes. The membranes were blocked with 5% non-fat milk in TNET buffer (10 mM Tris-Cl, pH7.5, 2.5 mM EDTA, 50 mM NaCl, 0.1% Tween-20) at RT for 1 h and blotted with primary antibodies overnight at 4 °C. After washing three times in TNET buffer, membranes were blotted in secondary antibodies at RT for 2 h. Chemiluminescent HRP substrate was applied on the membranes, and bands were visualized on a Bio-Rad ChemiDoc Imaging System.

### 2.4. Statistical Analyses

All data are presented as mean ± standard deviation unless otherwise noted. Two-tailed Student’s *t*-test was used to calculate *p*-values. *p*-values less than 0.05 were considered statistically significant.

## 3. Results

### 3.1. Embryonic Male Germ Cells Express Both Cul4 Genes

**Figure 1:**
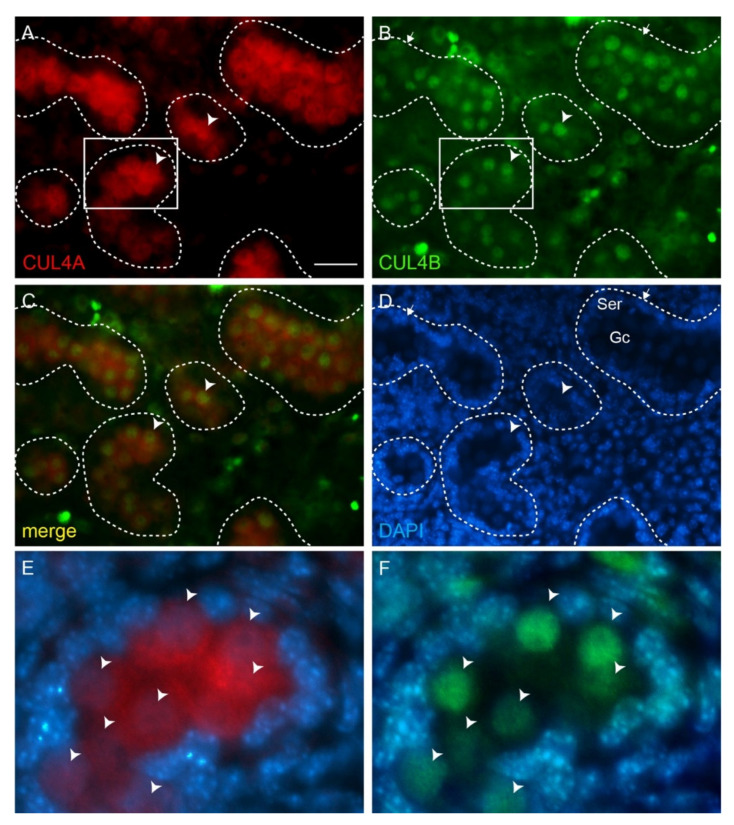
CUL4A and CUL4B co-localize in fetal gonocytes. (**A**–**F**) Immunofluorescence (IF) staining of CUL4A (red) and CUL4B (green) in E16.5 wild-type testis. Both CUL4A (**A**) and CUL4B (**B**) are highly expressed in the gonocytes (arrowheads), with CUL4B more prominent in the nuclei and CUL4A in both nuclei and cytoplasm. (**C**) shows merged CUL4A and CUL4B staining, and (**D**) shows DAPI counterstain. (**E**,**F**) are higher-magnitude views of the boxed areas in (**A**,**B**), respectively, with DAPI staining overlaid. Arrowheads point to gonocytes. Dashed lines outline seminiferous tubules. Ser, Sertoli cells; Gc, gonocytes. Scale bar 50 µm.

We have previously reported a dynamic and complimentary expression pattern of the two CUL4 proteins in the postnatal developing and adult mouse testes [11]. In the adult testis, CUL4A is predominantly expressed in primary spermatocytes, whereas CUL4B expression is prominent in spermatogonia, spermatids, and Sertoli cells [11]. As *Cul4b* is located on the X-chromosome, we believe that its absence in primary spermatocytes is caused by meiotic sex chromosome inactivation (MSCI), a transcriptional silencing process of the X and Y chromosomes that occurs during the meiotic phase of spermatogenesis [15]. Unlike in the adult testis, embryonic male gonocytes express both CUL4 proteins simultaneously. At embryonic day 16.5 (E16.5), CUL4A is detected in both the cytoplasmic and nuclear compartments of male gonocytes (Figure 1A,C–E arrowheads), and CUL4B is primarily detected in the gonocytes’ nuclei (Figure 1B–D,F arrowheads). CUL4B can be also detected in embryonic Sertoli cells, albeit at a much lower level (Figure 1B, arrows). 

### 3.2. Germ Cell-Specific Cul4a/4b-Double Null Males Lose All Germ Cells before Puberty

**Figure 2:**
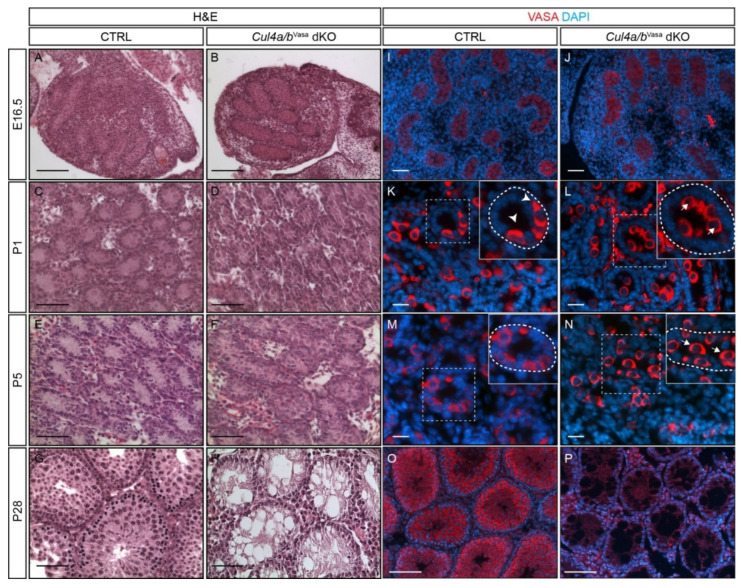
*Cul4* genes are essential for spermatogenic cell survival and germ cell homing. (**A**–**H**) Morphology of testes from E16.5, P1, P5 and P28 CTRL and *Cul4a/b*^Vasa^ dKO mice by H and E staining. Relative normal morphology was observed in neonatal dKO mutants, however, by P28 the mutant testes are filled with empty tubules. (**I**–**P**) IF staining of germ cell marker, VASA, in testicular sections of E16.5, P1, P5 and P28 CTRL and *Cul4a/b*^Vasa^ dKO mice. In neonate mutants, VASA-positive germ cells are present in the dKO seminiferous tubules, but show delay in homing. Insets in (**K**–**N**) show magnified view of boxed areas; dashed lines outline individual tubules. Note that clusters of germ cells are positioned in the mutant seminiferous tubule lumens (arrows, insets in **L**,**N**), whereas in the CTRLs they have migrated to the basement membranes (arrowheads, insets in **K**,**M**). By P28, VASA-positive germ cells are no longer detectable in dKO testes. Scale bars: 100 µm in (**A**–**D**), (**O**,**P**); 50 µm in (**E**–**N**).

Given the extensive sequence homology between the two *Cul4* genes and functional redundancy in cell culture (for review see [16]), we hypothesize that the two genes also function redundantly during gonocyte development. To test this hypothesis, we generated germ cell-specific *Cul4a/4b* double knockout mice using the well-established *Vasa*-Cre line [17]. *Vasa*-Cre mediates efficient and specific genomic recombination in the germ cell lineage as early as E15, and reaches > 95% efficiency by P0. *Vasa*-Cre transgenic mice were bred to *Cul4a*^f/f^ mice first, and *Vasa*-Cre+;*Cul4a*^f/+^ male progenies were crossed to *Cul4a*^f/f^;*Cul4b*^f/f^ females to obtain *Vasa*-Cre+;*Cul4a*^Δ/f^;*Cul4b*^f/Y^ double conditional knockout males (hereinafter referred to as *Cul4a/b*^Vasa^ dKO) and littermate controls (CTRL) (Supplementary Figure S1). *Cul4a/b*^Vasa^ dKO males develop normally as their littermate controls, but are completely sterile. Testes isolated from neonatal *Cul4a/b*^Vasa^ dKO mice were normal in size (CTRL, 11.3 ± 0.8 mg/testis, n = 4; dKO, 9.9 ± 1.1 mg/testis, n = 4; *p* = 0.081), but by P28 they were significantly smaller and weighed less than controls (Supplementary Figure S2**,** CTRL, 47.7 ± 2.0 mg/testis, n = 4; dKO, 14.9 ± 1.0 mg/testis, n = 4; *p* = 1.0 × 10 ^−7^. Histologically, the embryonic and neonatal mutant testes presented relatively normal morphology by P5 except prominent cell bodies in the lumen of seminiferous tubules of *Cul4a/b*^Vasa^ dKO mice (Figure 2A–F). However, by P28, when round and elongated spermatids started to emerge in the CTRL testes (Figure 2G), mutant seminiferous tubules only contained numerous vacuoles resembling the pathological characteristics of human Sertoli cell-only syndrome (Figure 2H). To monitor the progression of germ cells, immunofluorescence (IF) against the germ cell marker, VASA, was performed on testes sections (Figure 2I–P). At E16, one day after *Vasa*-Cre activation, both the CTRL and *Cul4a/b*^Vasa^ dKO seminiferous tubules were filled with VASA-positive gonocytes (Figure 2I,J). At P1, the majority of germ cells were still positioned in the lumen of the dKO seminiferous tubule (Figure 2L inset, arrows); whereas, in the CTRL testis, most gonocytes had migrated to the basement membrane of the tubules (Figure 2K inset, arrowheads). By P5, all gonocytes in CTRL testes had finished migration, while in contrast, many mutant germ cells still remained in the lumen (Figure 2N inset, arrows). By P28, no VASA-positive cells were ever detected in the *Cul4a/b*^Vasa^ dKO testis, indicating a complete loss of germ cells during the first wave of spermatogenesis (Figure 2P).

**Figure 3:**
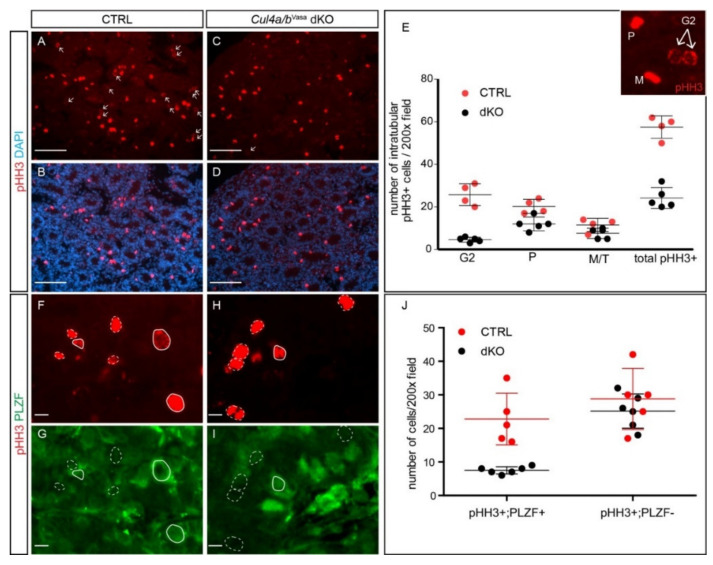
*Cul4* genes are critical to maintain male germ cell proliferation. (**A**–**D**) IF staining of phospho-histone H3 (pHH3, red) in P5 CTRL and *Cul4a/b*^Vasa^ dKO testes. Arrows point to pHH3-positive G2 phase cells. (**E**) Quantification of pHH3-positive cells in seminiferous tubules revealed significant reduction in number of pHH3+ cells in the dKO, particularly in cells at G2 phase. Inset shows typical pHH3 staining pattern of cells at prophase (P), metaphase (M) and G2 phase. M/T, metaphase/telophase. Total: CTRL 57.5 ± 5.3, dKO 24.0 ± 5.3, *p* = 2.5 × 10 ^−5^; G2: CTRL 25.8 ± 5.1, dKO 4.8 ± 1.3, *p* = 3.9 × 10 ^−5^; P: CTRL 20.2 ± 3.3, dKO 13.0 ± 2.7, *p* = 0.007; M/T: CTRL 11.5 ± 3.1, dKO 13.0 ± 2.7, *p* = 0.07; n = 4 for CTRL and n = 5 for dKO. (**F**–**I**) Double IF staining of pHH3 (red) and PLZF (green) of P5 CTRL and dKO testicular sections. Solid white lines circle out pHH3+; PLZF+ proliferating germ cells, dashed white lines circle out pHH3+; PLZF- cells. (**J**) Quantification of pHH3/PLZF double IF cells revealed significant decrease in number of double positive cells. pHH3+; PLZF+: CTRL 22.8 ± 7.7, dKO 7.5 ± 1.0, *p* = 8.8 × 10 ^−4^; pHH3+; PLZF−: CTRL 28.8 ± 9.1, dKO 25.1 ± 5.1, *p* = 0.42; n = 5 for CTRL and n = 6 for dKO. Scale bars: 50 µm in (**A**–**D**), 20 µm (**F**–**I**).

Extensive studies have demonstrated that CUL4-DDB1 ubiquitin ligase complex is crucial for cell cycle progression and cell survival [13,17,18,19,20]. The loss of germ cell phenotype prompted us to examine whether they play a similar role in regulating male gonocyte cell cycle progression. IF staining against phospho-histone H3 (pHH3), a proliferation marker that labels cells in G2-M phase, showed a significant reduction in pHH3+ cell number inside the dKO seminiferous tubules (Figure 3A–E, CTRL 57.5 ± 5.3, dKO 24.0 ± 5.3, *p* = 2.5 × 10 ^−5^). pHH3 has distinct staining patterns, indicative of various cell cycle stages: at the G2 phase, scattered pHH3 foci start to form at the nuclear periphery (Figure 3E inset, arrows); at the prophase, condensed and intensive pHH3 staining fill the whole nucleus (Figure 3E inset, P); at the metaphase, compacted pHH3 staining is detected at the equatorial plate (Figure 3E inset, M); and finally at the anaphase/telophase, pHH3 signals rapidly diminish as sister chromatins uncoil and histones are dephosphorylated. Noticeably, a closer examination and quantification revealed that the reduction in pHH3+ cells resides particularly in the G2 cell population of the mutant testis (Figure 3E, CTRL 25.8 ± 5.1, dKO 4.8 ± 1.3, *p* = 3.9 × 10 ^−5^). These cells have large, round and pale nuclei with prominent nucleoli morphology (Supplementary Figure S3, arrowheads), indicating that they are gonocytes. Indeed, double IF of pHH3 and gonocyte/undifferentiated spermatogonia marker, promyelocytic leukemia zinc finger (PLZF), confirmed that the number of proliferating PLZF+ gonocytes was significantly reduced in the mutant, whereas that of proliferating PLZF− somatic cells was indistinguishable between the CTRL and mutant seminiferous tubules (Figure 3F–J). These data demonstrate that the loss of both CUL4 proteins in the developing male germ cells compromised their ability to proliferate.

**Figure 4:**
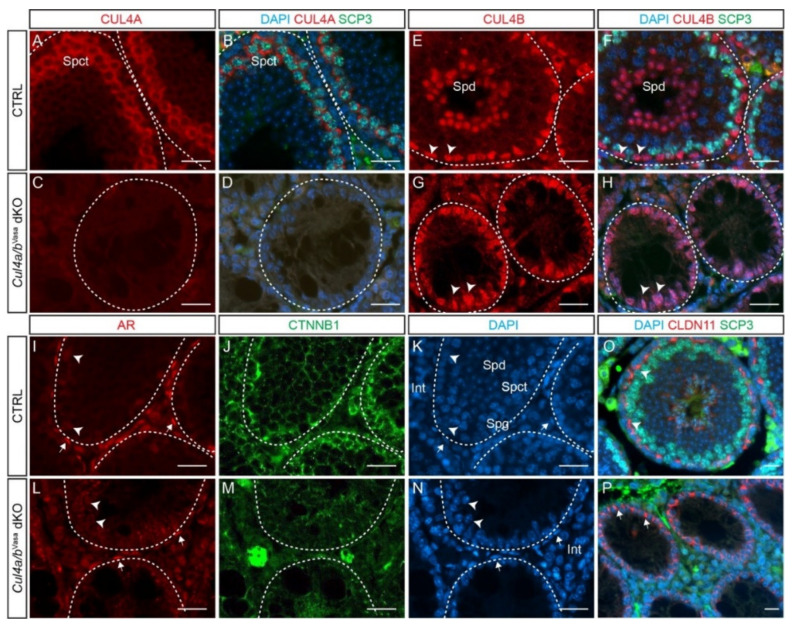
*Cul4* genes are required for male germ cell survival. (**A**–**D**) IF staining of CUL4A (red) and SCP3 (green) of P28 testis. While primary spermatocytes expressing both CUL4A (**A**) and SCP3 (**B**) are present in the CTRL testis, this population is completely missing in the dKO mutant testis (**C**,**D**). (**E**–**H**) IF staining of CUL4B (red) and SCP3 (green) of P28 testis. CUL4B is highly expressed in the Sertoli cells (**E**, arrowheads) and round spermatids (Spd) of CTRL testis (**E**,**F**); whereas in the *Cul4a/b*^Vasa^ dKO, CUL4B-positive Sertoli cells are the only cell type remaining in the seminiferous tubules (**G**,**H** arrowheads). (**I**–**N**) IF staining of AR (red) and CTNNB1 (green) of P28 testicular sections. Wild-type testis show AR-high myoid cells (**I**, arrows) and AR-low Sertoli cells (**I**, arrowheads), and well organized germ cell/Sertoli cell network outlined by CTNNB1 staining (**J**). The dKO mutant testis retains myoid and Sertoli cells (**L**, arrows and arrowheads, respectively), with disorganized residual CTNNB1 staining (**M**). DAPI counterstain is shown in (**K**) and (**N**). Spd, spermatids; Spct, spermatocytes; Spg, spermatogonia; Int, interstitial cells. (**O**,**P**) IF staining of CLDN11 (red) and SCP3 (green) of P28 testis. At this stage in the wildtype testis, tight junctions have formed apical to the spermatogonial stem cells to separate the basal and adluminal compartments (**O**, arrowheads). CLDN11 was only detected in the basal-lateral membranes of dKO Sertoli cells (**P**, arrows). Scale bars: 50 µm.

To better characterize the mutant testis phenotype at P28, IF of CUL4A, CUL4B and synaptonemal complex protein 3 (SCP3), a key component of the synaptonemal complex—which assembles only during prophase I [21] and is a marker for primary spermatocytes—was performed (Figure 4A–H). At P28, cytoplasmic CUL4A staining was exclusively detected in primary spermatocytes, marked by SCP3 staining (Figure 4A,B). Neither CUL4A nor SCP3 was detected in the mutant testes (Figure 4C,D). Nuclear CUL4B staining was detected in round spermatids and spermatogonia, as well as in Sertoli cells at P28 in the CTRL seminiferous tubules (Figure 4E,F); however, the mutant tubules retained only CUL4B-positive Sertoli cells. (Figure 4G,H). These data demonstrated the complete loss of male germ cells and confirmed the complete ablation of the two *Cul4* genes by *Vasa*-Cre in the mutant testes. To further evaluate the nature of the remaining cells in the *Cul4a/b*^Vasa^ dKO testis at P28, IF of androgen receptor (AR) and β-catenin (CTNNB1) was performed (Figure 4I–N). Strong AR signal was detected in the CTRL interstitial and peritubular myoid cells (Figure 4I, arrows) and in Sertoli cells, to a lesser extent (Figure 4I, arrowheads), which remained unchanged in the mutants (Figure 4L). β-catenin is reported to be expressed in Sertoli cells mainly on the membrane starting from E15.5 [22]. At P28, membrane β-catenin staining was evident in the CTRL testis, outlining the highly-organized Sertoli-germ cell network (Figure 4J). Disorganized β-catenin staining was detected in the mutant seminiferous tubules (Figure 4M). Loss of germ cells may also indicate a defective BTB, the junction network formed between adjacent Sertoli cells to create the SSC niche that separates the basal and adluminal compartments. Double IF staining of SCP3 and claudin 11 (CLDN11), a key component of tight junctions (TJs), showed apically located TJs separating the spermatogonia and SCP3+ primary spermatocytes (Figure 4O, arrowheads). Even though CLDN11 was also detected in the dKO testis, they are mostly basal-laterally located (Figure 4P, arrows), indicating a potential SSC niche defect. Taken together alongside our previous findings that genetic ablation of single *Cul4* gene in germ cells only causes defects in later-stage spermatogenesis [11,12], our present data demonstrates that the two CUL4 proteins play functional redundant but indispensable roles for male gonocyte homing, viability, and potentially for SSC niche establishment. 

### 3.3. Genetic Ablation of Cul4b in Both Sertoli and Germ Cells Leads to Seminiferous Tubule Structural Defects and Germ Cell Reduction

**Figure 5:**
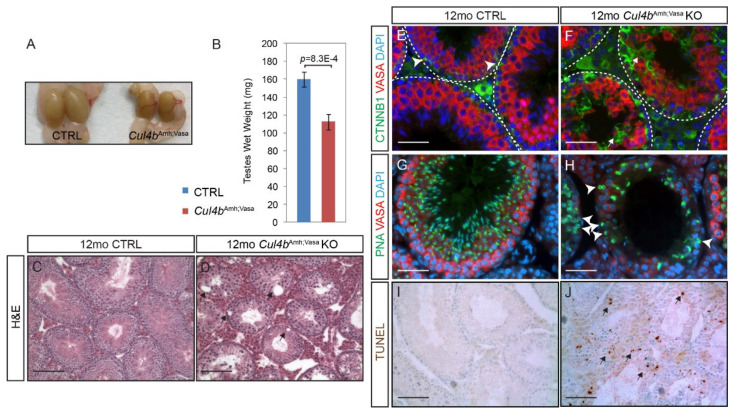
Ablation of *Cul4b* in both germ cells and Sertoli cells causes extensive germ cell death. (**A**) Representative gross morphology of *Cul4b*^Amh;Vasa^ KO and CTRL testes at 12 months. (**B**) Wet weight of testes isolated from 12mo mice. Data presented as mean ± s.d, n = 3 biologically independent testes. (**C**,**D**) Morphology of 12mo testicular sections by H and E staining. Mutant seminiferous tubule diameters are in general smaller, and many voids are visible inside the tubules indicating defective epithelial structure (arrows). (**E**,**F**) IF staining of VASA (red) and β-catenin (green). VASA-positive germ cells are orderly arranged, and β-catenin is most strongly expressed surrounding the basal-localized spermatogonia in the CTRL testis (**E**, arrowheads). Mutant germ cells are disorganized inside the tubules, and intensive β-catenin staining is detected throughout the length of Sertoli cells (**F**, arrows). (**G**,**H**) IF staining of VASA (red) and lectin PNA (green). PNA-positive spermatids are close to the lumen and positioned inside of the ring of VASA-strong primary spermatocytes, as spermatogenesis progresses in the CTRL testis. In the mutant, PNA-positive spermatids are substantially reduced in number, and many are abnormally positioned next to the basement membrane (**H**, arrowheads). (**I**,**J**) TUNEL-staining revealed extensive cell death inside the mutant seminiferous tubules (arrows). Scale bars: 200 µm in (**C**,**D**), 50 µm in (**E**–**H**), 100 µm in (**I**–**J**).

In our previous study, we have reported that germ-cell specific removal of *Cul4b* (*Cul4b*^Vasa^) led to male infertility due to spermiogenesis defects, whereas global *Cul4b* knockout mice (*Cul4b*^Sox2^) exhibited an age-dependent germ-cell depletion phenotype [12]. This discrepancy in phenotype suggests that CUL4B function is required during spermatogenesis in both stem cell maintenance/renewal and germ cell differentiation. Intriguingly, even though CUL4B is the only CUL4 protein expressed in Sertoli cells, its function is completely dispensable as conditional *Cul4b* ablation in these cells by *Amh*-cre did not result in any spermatogenesis defect [12]. To eliminate the possibility that cellular crosstalk between germ and Sertoli cells ensured normal spermatogenesis, we removed *Cul4b* from both cell populations (*Cul4b*^Amh;Vasa^ KO) and characterized its phenotype at 12 months old (12 mo). The mutants were obtained by first crossing male *Amh*-Cre transgenic mice with *Cul4b*^f/f^ females, then crossing *Amh*-Cre;*Cul4b*^f/+^ female progenies with *Vasa*-Cre male mice. Mutant testes at this age were significantly smaller and weighed less (Figure 5A,B CTRL, 159.9 ± 8.5 mg/testis, n = 3; *Cul4b*^Amh;Vasa^ KO, 112.3 ± 9.0 mg/testis, n = 3; *p* = 0.00083). To our surprise, the germ cell depletion phenotype exhibited by the global *Cul4b* null mutant [12] was not fully recapitulated in aged *Cul4b*^Amh;Vasa^ KO testes either. Histological analysis revealed the presence of vacuoles of various sizes in almost all the mutant seminiferous tubules, nevertheless, germ cells were present in all tubule sections (Figure 5D), even though in much fewer numbers compared to CTRL tubules (Figure 5C). VASA IF staining confirmed the presence of germ cells in the mutant tubules (Figure 5F). It is noteworthy that despite their presence, mutant germ cells were not as structurally organized as their CTRL counterparts (Figure 5E, red), and appeared desynchronized in development (Figure 5F and Supplementary Figure S4). Unlike the CTRL testis where each seminiferous tubule section can be readily staged based on the composition of distinct spermatogenic cell populations, mutant tubular sections cannot be staged due to the presence of germ cells of various developing steps that normally would not appear together. To facilitate illustrating intratubular structure, IF staining of β-catenin was performed. At this stage, β-catenin was most prominently detected at the apical side of spermatogonia located closed to the basement membrane (Figure 5E, green, arrowheads), with less intensity surrounding germ cells of later developmental stages as they progress toward the lumen. Markedly elevated β-catenin protein level was noted in the mutant tubules, highlighting the distorted Sertoli-germ cell network (Figure 5F, green, arrows). IF staining of lectin PNA (peanut agglutinin), which exclusively binds to the outer acrosomal membrane, was performed to assess spermatids progression. Substantially fewer PNA-positive spermatids were present in the mutant tubules (Figure 5H compared to Figure 5G). In addition, many PNA-positive round- or elongated-spermatids were present adjacent to the basement membrane of the mutant seminiferous tubules, a phenomenon rarely observed in the CTRL (Figure 5H, arrowheads). The significant loss of germ cells in the *Cul4b*^Amh;Vasa^ KO testis prompted us to examine cell death by TUNEL staining, and indeed massive numbers of apoptotic cells were detected in the mutant (Figure 5J, arrows). As a result, almost no mature spermatozoa were present in the mutant caudal epididymis (Supplementary Figure S5). Collectively these data demonstrate that genetic removal of *Cul4b* in both germ cells and Sertoli cells led to male infertility phenotypically distinct from that of the *Cul4b*^Amh^ and *Cul4b*^Vasa^ single knockout, as well as that of the global *Cul4b* null mutants.

### 3.4. CUL4B Is Required to Maintain BTB Integrity

**Figure 6:**
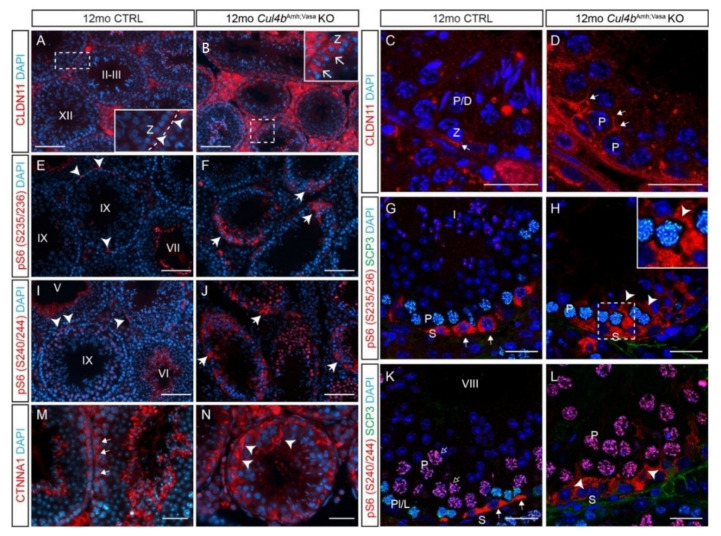
Ablation of *Cul4b* in both germ cells and Sertoli cells leads to BTB defects. (**A**,**B**) IF staining and (**C**,**D**) confocal microscopy of tight junction marker CLDN11 in CTRL and *Cul4b*^Amh;Vasa^ testis. The insets in A and B are magnified views of boxed areas. Basement membrane outlined by dashed lines in insets. (**E**,**F**) IF staining of pS6 (S235/236) showing its accumulation in the mutant tubules. (**G**,**H**) Confocal IF of pS6 (S235/236) (red) and SCP3 (green) showing localization of pS6 (S235/236) in CTRL spermatogonia (**G**, arrows), and ectopic activation in the mutant Sertoli cells (**H**, arrowheads). (**I**,**J**) IF staining of pS6 (S240/244) showing its accumulation in the mutant tubules. (**K**,**L**) Confocal IF of pS6(S240/244) (red) and SCP3 (green) showing its normal expression in spermatogonia (**K**, arrows) and ectopic activation in the mutant germ cells (**L**, arrowheads). (**M**,**N**) IF of α-catenin (CTNNA1) showing its accumulation in the mutant testis. S, spermatogonia; P, pachytene spermatocytes; Z, zygotene spermatocytes; Spg,. White dashed lines outline the seminiferous tubules. Scale bars: 200 µm in (**A**,**B**), (**E**,**F**), (**I**,**J**); 50 µm in the rest.

The appearance of basally positioned spermatids and the overall impaired tubule structure prompted us to speculate that the loss of *Cul4b* in the *Cul4b*^Amh;Vasa^ KO testis compromised the integrity of BTB. The BTB consists of several types of junctions: tight junctions (TJs) that are ubiquitously found in epithelial cells, and basal ectoplasmic specializations (ESs) and desmosome-gap junctions (D-GJs) that are unique to the testis [23]. Starting at around stage VIII of the epithelial cycle, the cohort of preleptotene spermatocytes near the basement membrane must traverse the BTB to continue meiosis in the adluminal compartment. This is accomplished by de novo synthesis and assembly of a “new” barrier below the migrating preleptotene spermatocyte, and dissociation of the “old” BTB. IF staining of the key TJ component, CLDN11, revealed cyclic TJ formation in the CTRL seminiferous tubules (Figure 6A). A high-magnification view of the boxed area shows, at stage XI, newly assembled tight junctions appeared beneath the zygotene spermatocytes, as they had exited the basal compartments (Figure 6A inset, arrowheads). Elevated CLDN11 staining, specifically in the cytoplasm of Sertoli cells, was detected in many mutant tubules (Figure 6B inset, arrows). Confocal IF microscopy further confirmed this finding (Figure 6C,D). Recent studies have shown evidence to support the critical involvement of mTOR (mammalian target of rapamycin) signaling in BTB dynamics, in that the mTORC1 complex appears to facilitate BTB remodeling and mTORC2 stabilizes it [24]. Intriguingly, mTORC1 function requires CUL4-DDB1 complex and Raptor, a central component of mTORC1 that is also a DDB1-CUL4 substrate [25]. Activation of mTORC1 is first signaled by phosphorylation of ribosomal protein S6 (rpS6) at Ser235/236 and Ser240/244 by S6 Kinase 1 [26]. In the CTRL testis, both phosphorylated forms of rpS6 were detected in the differentiated spermatogonia (Figure 6E,G,I,K, arrows). In addition, phosphorylated-rpS6 (pS6) at S240/244 was also detected in the nuclei of pachytene spermatocytes (Figure 6K, open arrows). Drastically elevated pS6 in both phosphorylation sites was detected in the mutant seminiferous tubules (Figure 6F,J,H,L, arrowheads). Close examination of the signal revealed that elevated pS6 proteins were primarily localized in the mutant Sertoli cells (Figure 6H,L note the voids surrounding the spermatocytes), indicating ectopic activation of mTORC1 in Sertoli cells. In addition to Claudins, another TJ-interacting structural protein, α-catenin, also abnormally accumulated in the mutant tubules (Figure 6M,N). Taken together, these data demonstrate that BTB dynamics are compromised in the absence of CUL4B, likely due to ectopically activated mTORC1 signaling pathway.

## 4. Discussion

In this study, we demonstrate that both CUL4 ubiquitin ligases are abundantly expressed by the gonocytes in the developing testis. Simultaneous inactivation of both *Cul4a* and *Cul4b* is detrimental to male gonocyte survival, as no spermatogenic cells remain in the *Cul4a/b*^Vasa^ dKO testis before the end of the first wave of spermatogenesis. In mammals, the two *Cul4* genes are coexpressed in many tissues and assemble structurally similar DDB-CUL4 complexes, which play essential roles in a variety of cellular functions including cell cycle progression, DNA damage repair and cell proliferation [27,28,29,30]. Due to their sequence homology and structural similarities, the two CUL4 proteins share many common substrates and often compensate for each other. Targeted inactivation of the CRL4 adaptor *Ddb1* (Damaged DNA Binding protein 1) caused early embryonic lethality in mice, and *Ddb1*-null mouse embryonic fibroblasts (MEFs) exhibited defects in cell growth and genomic stability [31]. Silencing of *Cul4b* in *Cul4a*-/- MEFs led to a dramatic reduction in cell proliferation and the loss of cell viability [13]. Our data provide further evidence that the CRL4 ligase activity is critical for cell survival, in the context of developing male germ cells.

One interesting finding of the *Cul4a/b*^Vasa^ dKO mutant is that the homing of gonocytes appeared to be delayed. In the mouse testis, gonocytes inside the seminiferous tubules migrate from the lumen towards the basement membrane shortly before birth, a process known as homing [5]. Successful homing relies on adhesion molecules and signaling molecules that are expressed by both gonocytes and Sertoli cells, such as c-*Kit*, β-integrin and *Sox8* [7,32,33]. Our current data demonstrate that the removal of both CUL4 proteins in germ cells leads to gonocyte homing delay, indicating the involvement of CUL4 substrates in this process. Their identities, however, remain unclear and demand further investigations. 

In our previous study, we reported that global abrogation of *Cul4b* leads to germ-cell depletion in aged mice, suggesting an involvement of CUL4B in SSC maintenance. However, removing *Cul4b*, specifically, in the germ cell population does not lead to this phenotype, despite spermiogenesis defects and male sterility; because *Cul4a* is not expressed in the adult spermatogonia, the distinctive male infertility phenotypes present in the global and germ cell-specific *Cul4b*–null mutants prompted us to speculate that a non-cell autonomous mechanism is responsible for CUL4B-mediated SSC maintenance. In an attempt to dissect the underlying mechanisms, we removed *Cul4b* from both germ and Sertoli cells in the current study and characterized its phenotype at 12 months of age. Surprisingly, the germ cell depletion phenotype was not detected in *Cul4b*^Amh;Vasa^ testes, either, suggesting a potential requirement of CUL4 function for SSC maintenance in other somatic cell populations, e.g., Leydig cells and residential testicular macrophages. Nevertheless, aged *Cul4b*^Amh;Vasa^ testes exhibited a novel phenotype not observed in the *Cul4b*^Vasa^ single mutant, where the BTB appeared to be defective. Fully differentiated spermatids that normally only reside in the adluminal compartment of the seminiferous epithelium, are often observed in the basal compartment of the *Cul4b*^Amh;Vasa^ testes, indicating a breach in the mutant BTB integrity. TJ marker CLDN11 IF staining further confirmed BTB impairment in the mutant, as considerably more intense staining was detected at the Sertoli interface apical to mid- to late-stage primary spermatocytes. Two possible scenarios could have led to this phenotype: (1) the BTB that separates the basal and adluminal compartments fail to dissemble in a timely manner, causing the spermatogonia to undergo differentiation within the basal compartment; and/or (2) the BTB has leakage which allows spermatocytes/spermatids to migrate back to the basal compartment. The dynamic assembly/disassembly of TJs is tightly controlled by the mTOR signaling pathway [24]. mTOR is a serine/threonine kinase that regulates many processes including transcription/translation, cell proliferation and survival. Depending on the binding factors, mTOR forms two complexes, mTORC1 and mTORC2, that exert distinct cellular functions. mTOR expression at the BTB is stage-specific, with the highest expression detected when the BTB undergoes active remodeling at stages VI-IX [34]. mTORC1 activation and the subsequent phosphorylation of rpS6 appear to promote BTB remodeling, making the barrier leaky; whereas the mTORC2 complex likely promotes BTB integrity through F-actin organization [35]. Fine-tuning the absolute and relative levels of mTORC1 and mTORC2 is achieved by many levels of regulation, among which is the ubiquitination of the central component of mTORC1, Raptor, by DDB1-CUL4 [25]. A downstream readout of the mTORC1 signaling pathway, phospho-rpS6 clearly showed a significant increase in abundance in the *Cul4b*^Amh;Vasa^ Sertoli cells, indicating ectopic activation of mTORC1. The fact that we observed marked elevation of CLDN11 in many mutant tubules, particularly in a diffused manner in the Sertoli cell cytoplasm, appears to support scenario 2, wherein the loss of CUL4B-mediated CRL4 ligase function leads to BTB dissembly via mTORC1 over-activation. Over time, this imbalance, favoring BTB breakdown, caused asynchronized germ cell development and BTB assembly/disassembly, eventually leading to the phenotype observed in the *Cul4b*^Amh;Vasa^ mutants. It is noteworthy that the Sertoli-specific *Cul4b*^Amh^ mutant does not exhibit such BTB defects; hence, it is plausible to conclude that CUL4B functions to mediate cell-to-cell crosstalk between Sertoli and germ cells to maintain BTB homeostasis and dynamics.

In summary, our current study provides genetic and functional data to demonstrate the indispensable role of CRL4 ligases for murine male gonocyte homing and survival, and its potential involvement in initial SSC niche establishment. We have also shown that CUL4B is required in both germ and Sertoli cells to maintain BTB homeostasis, likely through regulating the mTORC1 signaling activity. 

## Supplementary Materials

The following are available online at https://www.mdpi.com/article/10.3390/cells10102732/s1, Figure S1: Breeding scheme to generate *Vasa*-Cre+;Cul4aΔ/f;Cul4bf/Y double conditional knockout males. Figure S2: (A) Representative gross morphology of Cul4a/bVasa dKO and CTRL testes at P28. (B) Wet weight of testes isolated from P1 and P28 mice. Data presented as mean ± s.d, n = 4 biologically independent testes. Figure S3. Representative IF staining of pHH3. Figure S4. Germ cells in 12 month old Cul4bAmh;Vasa KO testis are disorganized and desynchronized. Figure S5. Histology of 12 month-old CTRL and Cul4bAmh;Vasa KO caudal epididymis.
