# Supplementary material for: Cullin4 E3 Ubiquitin Ligases Regulate Male Gonocyte Migration, Proliferation and Blood-Testis Barrier Homeostasis
Source: Cells. 2021 Oct 13;10(10):2732. doi: 10.3390/cells10102732 (PMC8535100; doi:10.3390/cells10102732)
Supplement: Supplementary file 1 [file cells-10-02732-s001.zip › cells-1386865-supplementary.pdf]

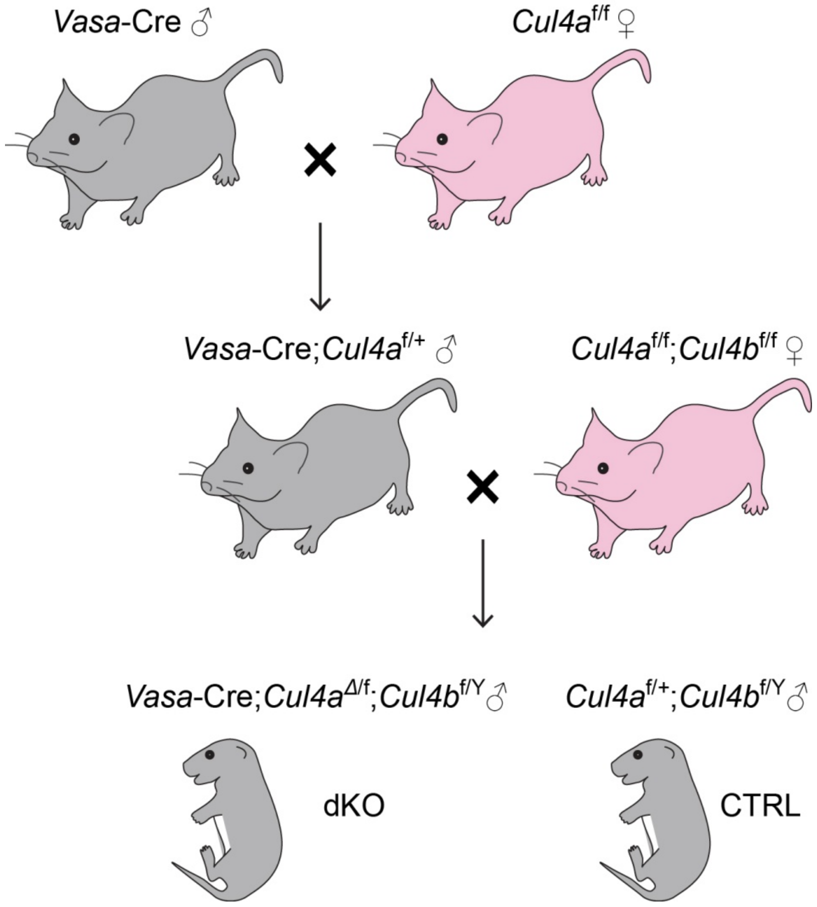

Figure S1. Breeding scheme to generate *Vasa*-Cre+;*Cul4a*$^{\Delta/f}$;*Cul4b*$^{f/Y}$ double conditional knockout males.

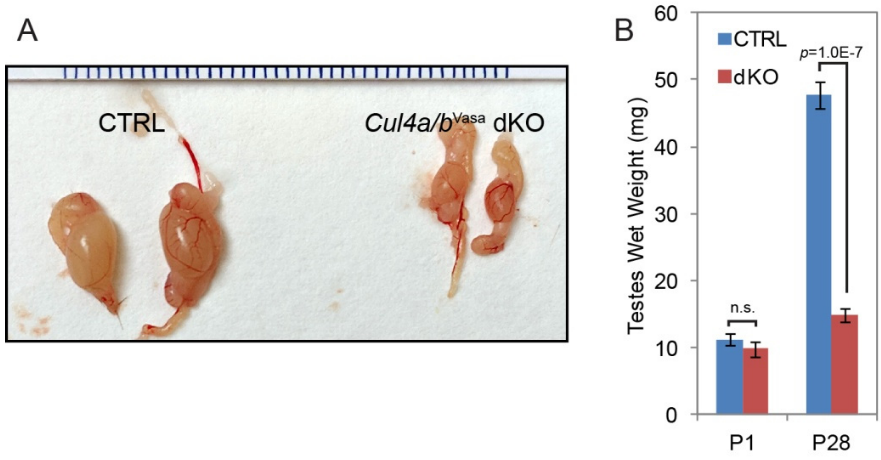

Figure S2. (A) Representative gross morphology of *Cul4a/b*^{Vasa} dKO and CTRL testes at P28. (B) Wet weight of testes isolated from P1 and P28 mice. Data presented as mean±s.d, n=4 biologically independent testes.

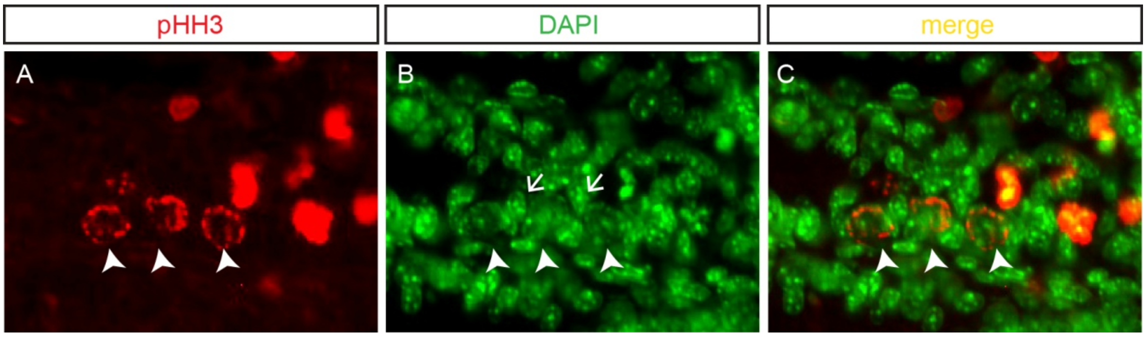

Figure S3. Representative IF staining of pHH3 (A) and nuclear morphology by DAPI staining (B) in WT P5 testis indicate that pHH3-positive G2 cells (arrowheads) are gonocytes with large, round and pale nuclei. Arrows point to Sertoli cell nuclei which are always oval-shaped.

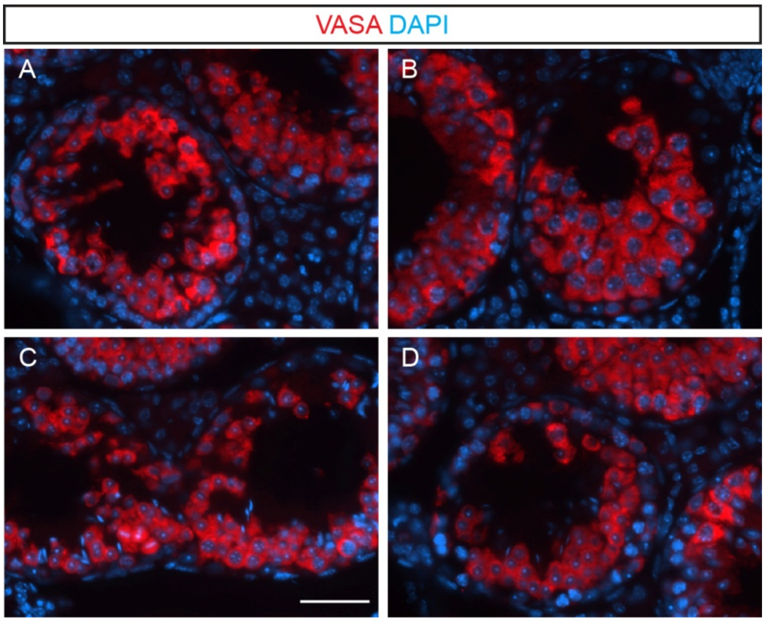

Figure S4. Germ cells in 12 month old *Cul4b*$^{Amh;Vasa}$ KO testis are disorganized and desynchronized. (A-D) Representative VASA IF images of KO seminiferous tubule sections. Unlike in the CTRL where very organized VASA-positive germ cells are present in every tubule sections in a stage-dependent pattern (Fig. 6E, G), KO germ cells are completely disorganized and appear to be desynchronized, and germ cells are missing in patches of regions within the tubules. Scale bar: 50 µm.

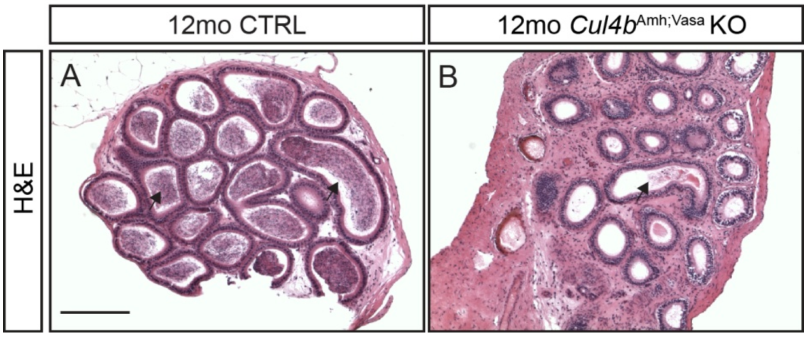

Figure S5. Histology of 12 month-old CTRL and *Cul4b*$^{Amh;Vasa}$ KO caudal epididymis. (A) Vast amount of spermatozoa are present in the lumen of CTRL epididymis (arrows). (B) mutant epididymis exhibited increased amount of interstitial tissue and greatly reduced spermatozoa inside the lumens (arrow). Scale bar: 200 µm.
